# A Multimodal Neuroimaging Study of Hyperlipidemia‐Induced Abnormal Spontaneous Brain Activity and Cognitive Decline

**DOI:** 10.1002/brb3.71890

**Published:** 2026-11-03

**Authors:** Jian‐Hui He, Jia‐Jia Wu, Fan‐Long Meng, Yun‐Ting Xiang, Rong‐Rong Huang, Mou‐Xiong Zheng, Jie Ma, Jian‐Guang Xu

**Affiliations:** ^1^ Department of Orthopedics and Traumatology Shuguang Hospital, Shanghai University of Traditional Chinese Medicine Shanghai People's Republic of China; ^2^ Department of Rehabilitation Medicine Yueyang Hospital, Shanghai University of Traditional Chinese Medicine Shanghai People's Republic of China; ^3^ School of Rehabilitation Science Shanghai University of Traditional Chinese Medicine Shanghai People's Republic of China; ^4^ Engineering Research Center of Traditional Chinese Medicine Intelligent Rehabilitation, Ministry of Education Shanghai People's Republic of China

**Keywords:** cognitive function, functional magnetic resonance imaging, hyperlipidemia, neuropsychological tests

## Abstract

**Objective:**

To investigate multidomain cognitive performance in older adults with hyperlipidemia and its associations with alterations in spontaneous brain activity and functional connectivity (FC).

**Methods:**

A total of 104 participants were recruited, comprising 50 hyperlipidemia patients and 54 healthy controls. Comprehensive psychological assessments were conducted, and resting‐state functional magnetic resonance imaging (rs‐fMRI) data were collected from both groups. Amplitude of low‐frequency fluctuations (ALFF), regional homogeneity (ReHo), and FC were calculated.

**Results:**

Compared with healthy controls, hyperlipidemia patients showed significantly lower SDMT scores (*p* < 0.001), whereas no significant between‐group differences were observed in the other neuropsychological measures. Imaging analyses revealed altered spontaneous brain activity and FC involving frontal, temporal, and cerebellar regions. In exploratory correlation analyses, lower SDMT scores and longer STT‐B completion times were associated with higher zALFF values in the right inferior temporal gyrus (*r* = −0.197, *p* = 0.046); however, the SDMT association was modest and did not survive correction for multiple comparisons (*r* = 0.257, *p* = 0.009). Age also positively correlated with zALFF in this region (*r* = 0.195, *p* = 0.048).

**Conclusions:**

Hyperlipidemia was associated with lower SDMT performance, altered spontaneous activity, and functional connectivity in the frontal, temporal, and cerebellar regions. The exploratory associations involving the right inferior temporal gyrus suggest a potential relationship between altered regional brain activity and cognitive performance.

## Graphical abstract

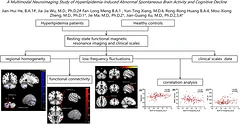

**Abbreviations**
ALFF: low‐frequency fluctuations
AVLT: Auditory Verbal Learning Test
BNT: Boston naming test
BOLD: blood oxygen level‐dependent
FC: functional connectivity
fMRI: functional magnetic resonance imaging
HAMD: Hamilton Depression Rating Scale
HDL: high‐density lipoprotein
IPS: information processing speed
LDL: low‐density lipoprotein cholesterol
MMSE: mini‐mental state examination
ReHo: regional homogeneity
rs‐fMRI: resting‐state fMRI
SDMT: Symbol Digit Modalities Test
STT‐B: Shape Trail Test
TC: total cholesterol
TMT: Trail Making Test

## 1 Introduction

Hyperlipidemia is a prevalent disorder of lipid metabolism, primarily characterized by elevated levels of low‐density lipoprotein cholesterol (LDL‐C), total cholesterol (TC), triglycerides (TG), and reduced levels of high‐density lipoprotein cholesterol (HDL) (T. J. Wang et al. 2019). It serves as a risk factor for atherosclerosis, contributing to high incidence and mortality rates of coronary heart disease and stroke (Asgary et al. 2018).

In recent years, increasing research has focused on the relationship between hyperlipidemia and cognitive function. Growing evidence suggests that hyperlipidemia can negatively impact brain function, particularly in areas such as learning, memory, and other cognitive functions (Y. Zhao et al. 2024). Clinical studies have shown that there is a significant correlation between high TC levels and poor cognitive function, and under the condition of a high TC value, cognitive function will become significantly worse (Thorvaldsson et al. 2020).

Hyperlipidemia has been associated with abnormalities in brain function, indicating that elevated cholesterol levels may result in reduced cerebral blood flow and neuronal damage, thereby impacting cognitive abilities and behavioral performance (Yoon et al. 2016). However, it remains uncertain whether alterations in cognitive function among hyperlipidemic patients correlate with their brain function abnormalities, and the underlying neurobiological mechanisms have yet to be elucidated.

Previous studies have demonstrated that dyslipidemia can directly remodel brain networks or modulate cerebrospinal fluid biomarkers, thereby triggering cognitive decline (Q. Wang et al. 2022). Population‐based research has identified significant correlations between triglyceride/apolipoprotein levels and cerebral structural abnormalities such as white matter hyperintensities (Han et al. 2025). Animal experiments further revealed that a high‐fat diet triggers behavioral deficits. MRI scans of BTBR mouse models detected lipid deposition in the fusiform gyrus, which was positively correlated with TC levels (Deng et al. 2026). Moreover, altered brain function has been linked to lipid profiles in patients with post‐stroke cognitive impairment and type 2 diabetes (X. Li et al. 2024). However, the neural mechanisms underlying cognitive alterations in patients with hyperlipidemia remain incompletely understood. In particular, it is unclear whether changes in spontaneous brain activity and functional connectivity (FC) are associated with performance across different cognitive domains. We hypothesized that patients with hyperlipidemia would exhibit altered resting‐state brain activity and FC, accompanied by differences in cognitive performance. Therefore, this study combined multidomain neuropsychological assessments with ALFF, ReHo, and seed‐based FC analyses to characterize hyperlipidemia‐related functional brain alterations and explore their associations with cognitive measures.

Specifically, several critical research gaps need to be addressed: (1) the specific mechanisms underlying how dyslipidemia impairs brain function (Chou et al. 2022); (2) the relationship between cognitive performance and brain function in patients with hyperlipidemia; and (3) the confounding factors that jointly modulate cognition and brain function among hyperlipidemic populations.

In neuroscience and cognitive science, functional magnetic resonance imaging (fMRI) serves as a valuable tool for studying brain function, allowing for non‐invasive monitoring of brain activity. This technique relies on measuring blood oxygen level‐dependent (BOLD) signals (Damoiseaux et al. 2006). In our study, three rs‐fMRI‐based biomarkers were measured to reflect local neural activity and FC in the brain. These measurements included the amplitude of low‐frequency fluctuations (ALFF), regional homogeneity (ReHo), and FC in resting‐state fMRI (rs‐fMRI). They are used to study the intensity of neural activity, local synchrony, and functional interactions between brain regions. These indicators help reveal the organization and potential abnormalities of brain functional networks, particularly in the study of neuropsychological and psychiatric disorders.

## 2 Methods

### 2.1 Participants

A total of 50 hyperlipidemic patients (24 males and 26 females) and 54 healthy controls (13 males and 41 females) were recruited for analysis. No patient was removed due to incomplete clinical data or MRI image artifacts. In this study, a group of hospitalized hyperlipidemia patients was selected from the Yueyang Hospital, affiliated with the Shanghai University of Traditional Chinese Medicine. The diagnosis was made by doctors from the Rehabilitation Department before the MRI scan. The diagnostic criteria were based on blood lipid levels, clinical symptoms, and routine blood tests, following Western diagnostic standards for hyperlipidemia. Hyperlipidemia was defined by hypercholesterolemia within the past six months. The inclusion criteria for hyperlipidemia patients were as follows: (1) aged between 40 and 80 years; (2) TC levels ranging from ≥ 5.20 to ≤ 6.20 mmol/L and LDL‐C levels ranging from ≥ 3.40 to ≤ 4.10 mmol/L (Okusaga et al. 2013); and (3) newly diagnosed hypercholesterolemia, with all data collection and MRI scans conducted before the initiation of any medication. The exclusion criteria for all participants were: (1) coexisting psychiatric disorders, poor compliance, or inability to cooperate with the study, such as dementia, severe cognitive impairment, or intellectual disability; (2) diagnosed or suspected cases of diabetes, cervical spondylosis, rheumatic diseases, or a history of brain disorders; (3) systemic peripheral neuropathy; and (4) contraindications for MRI, such as metallic implants, pacemakers, or severe claustrophobia. The study was approved by the local ethics committee of Yueyang Hospital, affiliated with the Shanghai University of Traditional Chinese Medicine. All individuals involved in the study gave written informed consent, in full accordance with the ethical guidelines outlined in the Declaration of Helsinki, ensuring their participation was voluntary and well‐informed.

### 2.2 Neuropsychological Evaluation

Attention was evaluated using the Symbol Digit Modalities Test (SDMT) (Fellows and Schmitter‐Edgecombe 2019). Emotional state was assessed using the Hamilton Depression Rating Scale (HAMD) and the Hamilton Anxiety Rating Scale (HAMA) (Rodriguez‐Seijas et al. 2020). Cognitive function was assessed using the Mini‐Mental State Examination (MMSE) (Y. Li et al. 2016). Executive function was measured with the Shape Trail Test (STT‐B) (Q. Zhao et al. 2013). Memory function was evaluated with the Auditory Verbal Learning Test (AVLT). Language function was assessed using the Boston Naming Test (BNT) (Tombaugh and Hubley 1997). Three senior neuropsychologists, each with over 12 years of work experience, conducted neuropsychological evaluations of the participants. One senior neuropsychology expert subsequently reviewed the assessment results. All individuals completed the neuropsychological evaluations.

#### 2.2.1 SDMT

Cognitive assessment was conducted using the SDMT. The SDMT is a neurophysiological test that evaluates information processing speed (IPS), visual scanning, and task memory (Fellows and Schmitter‐Edgecombe 2019). The test includes a key at the top of the page with nine symbols and their corresponding numbers (1–9). Below the key, there is a row of symbols arranged in a random order. The higher the score, the better the subject's cognitive function, particularly in terms of IPS and attention.

#### 2.2.2 HAMA and HAMD

The HAMA is one of the most widely used scales for assessing the severity of anxiety (Rodriguez‐Seijas et al. 2020). The scale includes 14 items, and each item is scored on a 5‐point scale ranging from 0 to 4: (0) no symptoms, (1) mild symptoms, (2) moderate symptoms, (3) severe symptoms, and (4) very severe symptoms. According to data provided by the American National Collaborating Committee on Scales, a total score exceeding 29 points may indicate severe anxiety; over 21 points suggests significant anxiety; over 14 points confirms anxiety; over 7 points indicates possible anxiety; and a score below 7 suggests no anxiety symptoms. Typically, the cut‐off score for the 14‐item version of the HAMA is set at 14 points. The total score of the HAMA reflects the severity of anxiety symptoms, with higher scores indicating more severe symptoms.

HAMD was first introduced by Hamilton and is widely used clinically to assess the severity of depression (Rodriguez‐Seijas et al. 2020; Zimmerman et al. 2013). Most items in the scale use a 5‐point scoring system: (0) no symptoms, (1) mild symptoms, (2) moderate symptoms, (3) severe symptoms, and (4) very severe symptoms. Several items are evaluated using a 3‐point scale, ranging from 0 to 2, with the criteria specified as follows: (0) no symptoms, (1) mild to moderate symptoms, and (2) severe symptoms, representing increasing levels of severity. For the 24‐item version of the assessment, a total score exceeding 35 points may indicate the presence of severe depression, while a score above 20 points suggests mild to moderate depressive symptoms, and a score below 8 shows no depression. A higher HAMD score indicates more severe depressive symptoms in the patient, reflecting an increased level of emotional distress.

#### 2.2.3 STT‐B

The STT‐B is a neuropsychological assessment tool used to evaluate executive functions, particularly cognitive flexibility, processing speed, and attention. It serves as an alternative to the traditional Trail Making Test (TMT), reducing the influence of language and educational background on performance. In the STT‐B, participants are required to connect a series of shapes as quickly and accurately as possible according to specific rules. This task assesses their ability to shift attention, process visual information, and execute complex visuomotor coordination (Q. Zhao et al. 2013). In the STT‐B, subjects need to connect a sequence of figures as quickly and accurately as possible following specific rules. Numbers are included within two types of shapes, squares, and circles, and when connecting the numbers in order, these two shapes must be alternated. Higher scores on the STT‐B correspond to worse cognitive function among subjects.

#### 2.2.4 AVLT

The AVLT is a neuropsychological tool designed to assess verbal memory, particularly auditory learning and retention (Can et al. 2016). The AVLT measures immediate recall, learning capacity, delayed recall, and recognition. Participants are presented with a fixed list of words (usually 15) multiple times, and are asked to recall as many words as possible after each presentation. Typically, five trials are conducted to evaluate memory acquisition and forgetting patterns. Afterward, an interference task may be introduced, followed by a delayed recall test. Finally, a recognition test is administered, requiring participants to identify words from the original list among distractors. The higher the AVLT score, the stronger the subject's memory and learning abilities are.

#### 2.2.5 MMSE

It is one of the most influential standardized tools for assessing mental status (Tombaugh and McIntyre 1992). It mainly evaluates the following cognitive functions: orientation (assessing awareness of time and place, 10 points), registration (requiring the participant to recall and instantly repeat three words, 3 points), attention and calculation abilities (e.g., requiring the subject to subtract 7 consecutively from 100 or spell a word backward, 5 points), recall (asking the subject to recall the three words previously learned, 3 points), language (evaluating naming, repetition, following three‐step commands, reading, writing, etc., 9 points), and visuospatial ability (requiring the subject to draw a simple figure, such as two overlapping pentagons, 1 point). The scoring criteria are as follows: the total score is 30 points. Generally, lower scores indicate poorer cognitive function. A score ranging from 24 to 30 indicates normal cognitive function, while 19 to 23 points suggest mild cognitive impairment, 10 to 18 points reflect moderate impairment, and a score of 0 to 9 indicates severe cognitive decline. A higher MMSE score signifies better cognitive health.

#### 2.2.6 BNT

It is a neuropsychological tool widely used to assess language function, specifically naming ability (Tombaugh and Hubley 1997). The specific testing process is as follows: before the test, participants will receive a brief demonstration task to ensure they understand the requirements and procedures of the test. Then, participants are presented with images of objects and are asked to name the objects as accurately as possible. The score is mainly recorded based on the number of correct answers given by the participant. Each correct response earns one point, and the overall score varies from 0 to 30 points, reflecting performance levels. A high BNT score usually indicates better language naming ability, better cognitive function, and a lesser degree of impairment.

### 2.3 FMRI Acquisition

The fMRI images were collected primarily in the mornings. Hyperlipidemia patients and healthy controls followed the same scanning protocol. In our experiment, we used data collection methods that employ a Siemens MAGNETOM Verio 3.0 T MRI system (Germany). Before scanning, participants were informed about precautions, such as avoiding metal implants or wearing metal items. During the MRI scan, participants were asked to remain still and close their eyes to rest, and avoid thinking or falling asleep. Foam pads were provided to reduce head movement.

The echo‐planar imaging parameters were—repetition time (TR) = 3000 ms; echo time (TE) = 30 ms; field of view (FOV) = 256 mm × 256 mm; slice thickness = 3 mm; number of slices = 43; matrix size = 64 × 64; total time points = 200; voxel size = 3.6 mm × 3.6 mm × 3 mm; and flip angle = 90°. The T1 sequence parameters were—TR = 1900 ms; inversion time = 900 ms; TE = 2.93 ms; flip angle = 9°; matrix size = 256 × 256; and slice thickness = 1 mm; all settings ensured optimal image resolution and quality.

### 2.4 FMRI Preprocessing

Our data were preprocessed using SPM12 and Restplus 1.25 software on the MATLAB 2022b platform. First, all the DICOM images were converted into Nifti format, and non‐brain tissues were excluded using Mricron software. The initial 10 time points were removed to minimize any impact from unstable MRI signals. Subsequently, slice timing correction was applied to the data. Motion correction was then performed, and the images were normalized to standard space using a standard template. In addition, detrending and covariate removal were applied to the data. To calculate the amplitude of ALFF, the images were smoothed to improve the signal‐to‐noise ratio. For ReHo calculation, temporal filtering was applied in the frequency range of 0.01–0.08 Hz to remove low‐frequency drifts and reduce high‐frequency noise effectively. We regressed out potential confounding variables, consisting of cerebrospinal fluid signals, white matter signals, and the 24 Friston head motion parameters. Participants showing translational head movement over 2 mm or rotational head movement exceeding 2° were excluded from further analyses (Chen et al. 2023).

### 2.5 ALFF Calculation

The ALFF represents the BOLD signal for each voxel. After preprocessing, ALFF was calculated using Restplus 1.25 software. This calculation occurred before filtering, with a frequency band of 0.01–0.08 Hz. The process involved applying a Fast Fourier Transform (FFT) to convert each voxel's time series into the frequency domain, obtaining the power spectrum, and then calculating its square root. The resulting values within the 0.01–0.08 Hz range were averaged to derive the ALFF value. The global mean ALFF was subtracted, and each subject's ALFF value was divided by the standard deviation (SD) of ALFF across all voxels, generating a zALFF map for every individual.

### 2.6 ReHo

After preprocessing, Restplus 1.25 software was used to calculate ReHo. Smoothing was not performed during preprocessing, but was applied after ReHo calculation. Kendall's coefficient of concordance (Kcc) was then computed to generate ReHo brain maps. KccReHo assesses time series consistency among voxels within a local brain region by calculating the Kcc, which reflects the synchronization of brain activity. Compared to the original ReHo method, KccReHo uses Kendall's coefficient to evaluate time series similarity, providing a more precise assessment of local functional activity synchronization (Zang et al. 2004).

### 2.7 FC

Based on the findings from the ALFF and ReHo analyses, we pinpointed significant changes of interest to serve as seed points. Therefore, the above brain regions were defined as regions of interest (ROIs), with a total of 10 ROIs, namely the right inferior temporal gyrus, the right middle temporal gyrus, the right middle temporal pole, the triangular part of the right inferior frontal gyrus, the right superior temporal pole, the dorsolateral right superior frontal gyrus, the medial right superior frontal gyrus, the medial left superior frontal gyrus, and two regions of the left inferior cerebellum. Masks of all 10 ROIs were extracted from the Automated Anatomical Labeling (AAL) atlas. The FC was calculated using Restplus 1.25 software. Fisher's z transformation was performed on the original connectivity matrices to generate Z‐maps, and standardized zFC values were acquired for subsequent statistical analyses. For statistical inference, two‐sample *t*‐tests were applied to identify intergroup differences in FC, with a primary threshold of *p* < 0.05. Family‐wise error (FWE) correction was applied at the cluster level to efficiently control false‐positive findings.

### 2.8 Statistical Analysis

We used SPSS 27.0 to analyze statistical differences in behavioral data between hyperlipidemia patients and healthy controls. Normality tests were first performed on raw data; two‐sample *t*‐tests were adopted for normally distributed data, while Mann‐Whitney *U* tests were used for non‐normal data. For imaging data, Restplus 1.25 software was applied to conduct statistical analyses of ALFF, ReHo, and FC. Two‐sample *t*‐tests were used to compare imaging metrics between the two groups, with FWE correction at the threshold of *p* < 0.005. Pearson correlation analyses were further carried out to examine the correlations between ALFF/ReHo values in significant brain clusters and clinical scale scores.

## 3 Results

### 3.1 Clinical Information

No significant between‐group differences were found in age, education level, height, weight, AVLT, STT‐B, SDMT, HAMA, HAMD, MMSE, or BNT scores (all *p* > 0.05, Table 1). However, the hyperlipidemia group had significantly lower SDMT scores than the healthy control group (*p* < 0.001).

**TABLE 1:** Basic Information of healthy controls and hyperlipidemia patients.

Parameter	Healthy controls	Hyperlipidemia patients	*p*‐value
	Mean ± SD	Mean ± SD	
Age (years)	65.56 ± 7.45	67.67 ± 5.81	0.354
Education (years)	10.62 ± 2.67	11.12 ± 2.68	0.352
Height (cm)	164.52 ± 7.40	162.42 ± 7.26	0.406
Weight (kg)	63.72 ± 10.31	64.18 ± 9.74	0.321
AVLT	26.30 ± 7.79	25.74 ± 9.66	0.746
STT‐B	140.10 ± 54.46	161.58 ± 82.75	0.118
SDMT	37.50 ± 9.49	29.58 ± 10.91	< 0.001^*^
HAMA	2.70 ± 2.24	2.68 ± 2.12	0.956
HAMD	1.11 ± 1.63	1.00 ± 1.53	0.721
MMSE	27.17 ± 1.65	27.26 ± 2.17	0.805
BNT	23.65 ± 2.62	23.24 ± 3.52	0.502

Abbreviations: AVLT, Auditory Verbal Learning Test; BNT, Boston Naming Test; HAMA, Hamilton Anxiety Rating Scale; HAMD, Hamilton Depression Scale; MMSE, Mini Mental State Examination; SDMT, Symbol Digit Mode Test; STT‐B, scores from Shape Trajectory Test B.

^*^There is a significant difference between the healthy control group and the hyperlipidemia group (*p* < 0.05).

### 3.2 ALFF

Compared to the healthy control group, patients in the hyperlipidemia group showed significantly increased zALFF values in the right dorsolateral superior frontal gyrus, the right medial superior frontal gyrus, the right triangular part of the inferior frontal gyrus, the right temporal pole of superior temporal gyrus, the right inferior temporal gyrus, the right middle temporal gyrus, the left medial superior frontal gyrus, and the right temporal pole of the middle temporal gyrus (FWE corrected, cluster‐level *p* < 0.005; Figure 1, Table 2 ).。

**FIGURE 1:**
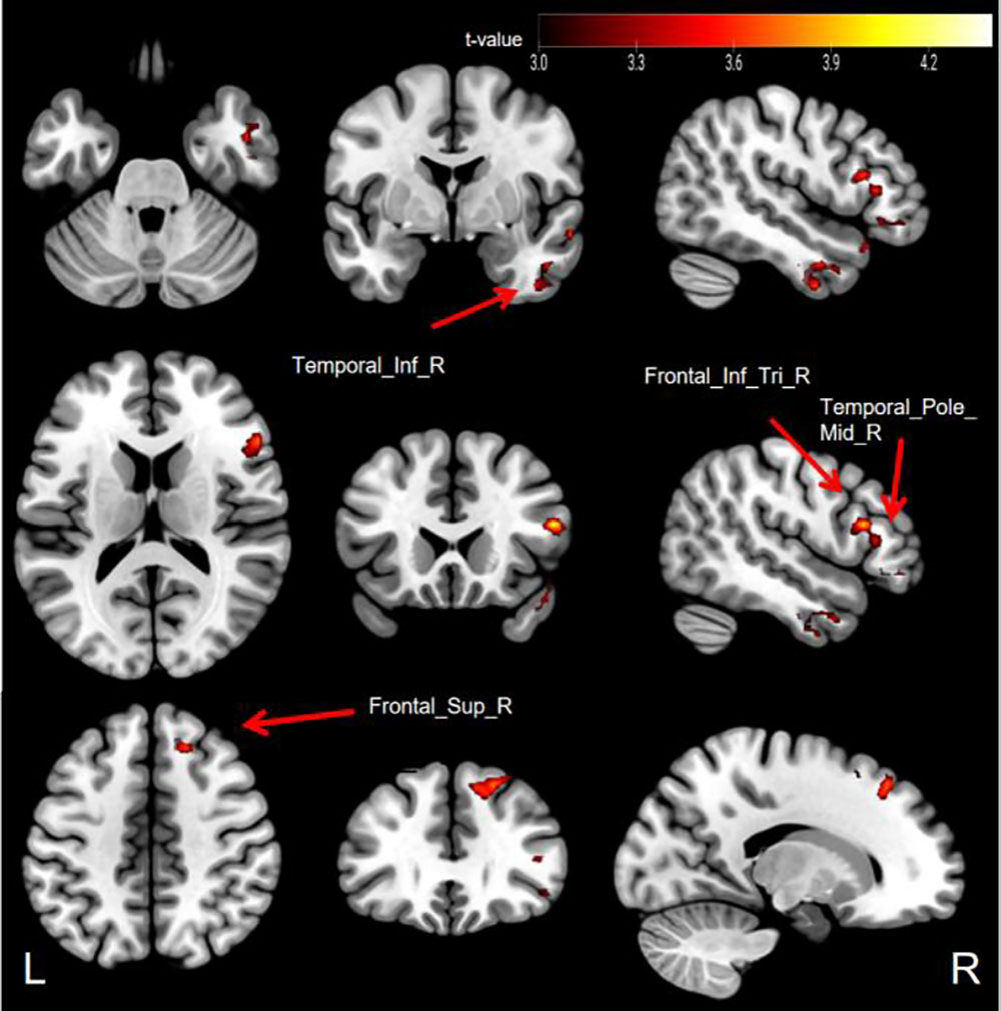
Comparison of amplitude of low frequency fluctuation (ALFF) between the hyperlipidemia group and the healthy control group. The figure demonstrates the results of comparison of zALFF values between two groups, with warmer colors indicating higher *t*‐value while comparing the hyperlipidemia group to the healthy group (FWE correction, cluster‐level *p* < 0.005). Temporal_Inf_R, right inferior temporal gyrus; Frontal_Inf_Tri_R, right inferior Frontal Gyrus of the Triangular Part; Frontal_Sup_R, right dorsolateral Superior Frontal Gyrus; L, left; R, right.

**TABLE 2:** Brain regions with increased zALFF while comparing the hyperlipidemia group to the healthy control group.

Contrast	Region label	Extent	*t*‐value	MNI coordinates		
				*x*	*y*	*z*
PT > HC	Temporal_Inf_R	201	4.667	42	15	−42
	Temporal_Mid_R	52	3.973	60	0	−15
	Temporal_Pole_Mid_R	42	4.667	42	15	−42
	Frontal_Inf_Tri_R	129	4.275	42	24	−27
	Temporal_Pole_Sup_R	43	3.608	54	12	−9
	Frontal_Sup_R	91	4.124	21	33	51
	Frontal_Sup_Medial_R	35	3.183	21	18	66
	Frontal_Sup_Medial_L	25	3.174	−9	30	51

Abbreviations: ALFF, amplitude of low frequency fluctuation; Frontal_Inf_Tri_R, right inferior frontal gyrus of the triangular part; Frontal_Sup_Medial_L, left medial superior frontal gyrus; Frontal_Sup_Medial_R, right medial superior frontal gyrus; Frontal_Sup_R, right dorsolateral superior frontal gyrus; HC, healthy control; MNI, Montreal Neurological Institutes; PT, hyperlipidemia patients; Temporal_Inf_R, right inferior temporal gyrus; Temporal_Mid_R, right middle temporal gyrus; Temporal_Pole_Mid_R, right temporal pole of middle temporal gyrus; Temporal_Pole_Sup_R, right temporal pole of Superior temporal gyrus.

### 3.3 ReHo

The KccReHo values in two inferior cerebellar regions of the hyperlipidemia group were significantly lower than those of the healthy control group (FWE correction, cluster‐level *p* < 0.005). These findings are presented in Table 3 and Figure 2.

**TABLE 3:** Brain regions with decreased KccReHo while comparing the hyperlipidemia group to the healthy control group.

Contrast	Region label	Extent	*T*‐value	MNI coordinates		
				*x*	*y*	*z*
PT < HC	Cerebelum_8_L	83	3.3601	−24	−51	−51
	Cerebelum_Crus2_L	24	3.206	−48	−63	−51

Abbreviations: Cerebelum_8_L, left inferior cerebellum; Cerebelum_Crus2_L, left inferior cerebellum; HC, healthy control; kccReHo, Kendall coefficient of concordance; MNI, Montreal Neurological Institutes; PT, hyperlipidemia patients.

**FIGURE 2:**
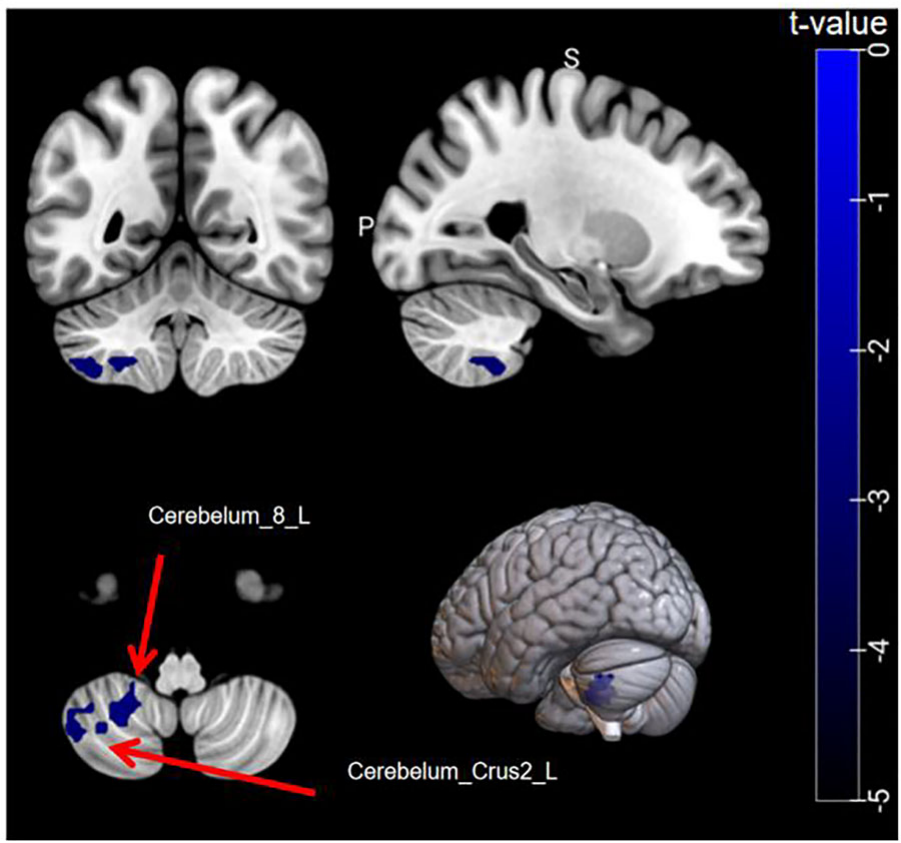
The comparison of regional homogeneity (ReHo) results between the hyperlipidemia group and the healthy control group. This figure demonstrates decreased kccReHo values in the hyperlipidemia group compared to the healthy group. The darker the color, the smaller the *t*‐value. (FWE correction, cluster‐level *p* < 0.005). Cerebelum_8_L, left inferior cerebellum; Cerebelum_Crus2_L, left Cerebellum_Inferior; L, left; R, right.

### 3.4 FC

Among all 10 predefined ROI seeds, only the dorsolateral right superior frontal gyrus exhibited significant intergroup differences. Compared with healthy controls, patients with hyperlipidemia showed significantly increased FC between this seed region and the right fusiform gyrus, the superior cerebellum, and the right middle temporal gyrus. The cluster contained 174 voxels with *t* = 3.849, and the significance survived FWE correction (*p* < 0.005) (Table 4, Figure 3, and Figure 4).

**TABLE 4:** Brain regions with enhanced functional connectivity (FC) between these brain regions with right dorsolateral superior frontal gyrus while comparing the hyperlipidemia group to the healthy control group.

Contrast	Region label	Extent	*T*‐value	MNI coordinates		
				*x*	*y*	*z*
PT > HC	Fusiform_R	174	3.849	39	−54	−18
	Cerebelum_4_5_R	57	3.826	21	−42	−15
	Temporal_Mid_R	10	3.247	60	−54	−3

Abbreviations: Cerebelum_4_5_R, right superior cerebellum; Fusiform_R, right fusiform gyrus; HC, healthy control; MNI, Montreal Neurological Institutes; PT, hyperlipidemia patients; Temporal_Mid_R, right middle temporal gyrus.

**FIGURE 3:**
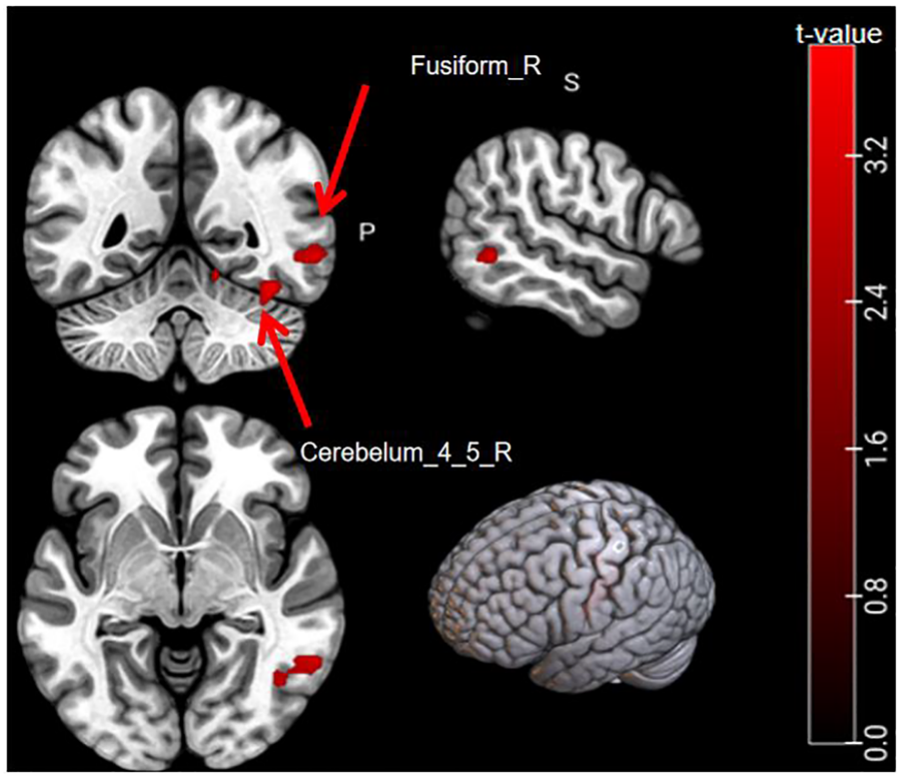
The comparison functional connectivity (FC) with dorsolateral superior frontal gyrus between the hyperlipidemia group and the healthy control group. Warm colors indicate brain regions with increased FC with the dorsolateral superior frontal gyrus, with warmer colors indicating higher *t*‐value while comparing the hyperlipidemia group to the healthy group (FWE correction, cluster‐level *p* < 0.005). Fusiform_R, right fusiform gyrus; Cerebelum_4_5_R, right cerebellum Superior; Temporal_Mid_R, right middle temporal gyrus.

**FIGURE 4:**
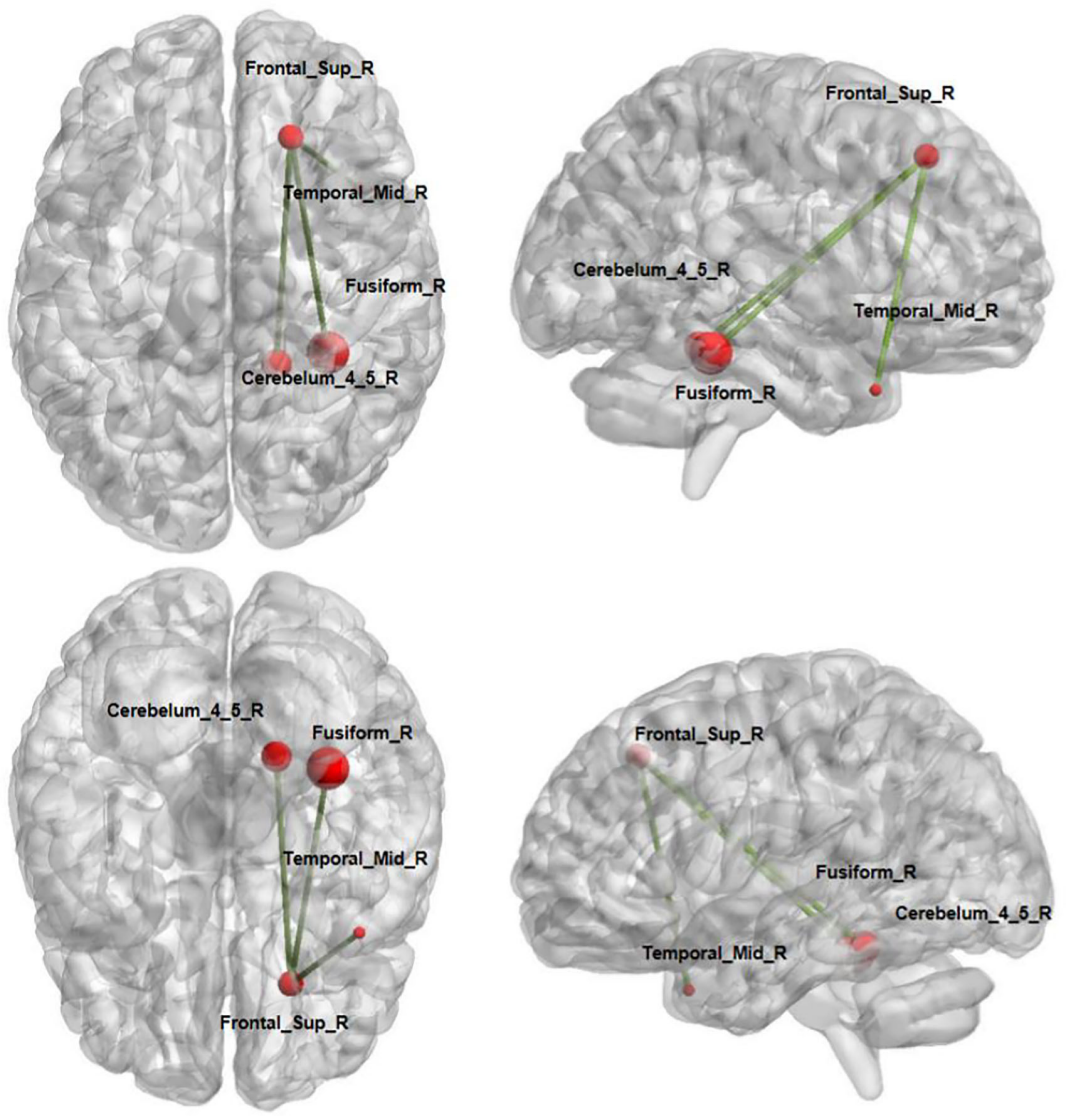
Functional connectivity (FC) maps of the superior dorsolateral frontal gyrus between the hyperlipidemia group and healthy control group. Fusiform_R: right fusiform gyrus; Cerebelum_4_5_R: right superior cerebellum (lobules 4 and 5); Temporal_Mid_R: right middle temporal gyrus.

### 3.5 Correlation Analysis

In the hyperlipidemia patients, the Pearson correlation analysis indicated positive correlation between age and the zALFF value of the right inferior temporal gyrus (*r* = 0.195, *p* = 0.048, uncorrected). A negative correlation was found between the SDMT and the zALFF values of the right inferior temporal gyrus (*r* = −0.197, *p* = 0.046, uncorrected), while STT‐B exhibits a positive correlation with the zALFF value in the same area (*r* = 0.257, *p* = 0.009, uncorrected). No significant correlation was found between the ReHo value in the right inferior cerebellar region or the clinical assessment scales. Furthermore, changes in FC among the right dorsolateral superior frontal gyrus, the right fusiform gyrus, the superior cerebellar region, and the right middle temporal gyrus showed no significant correlation with resting‐state FC or other clinical assessments (Figure 5).

**FIGURE 5:**
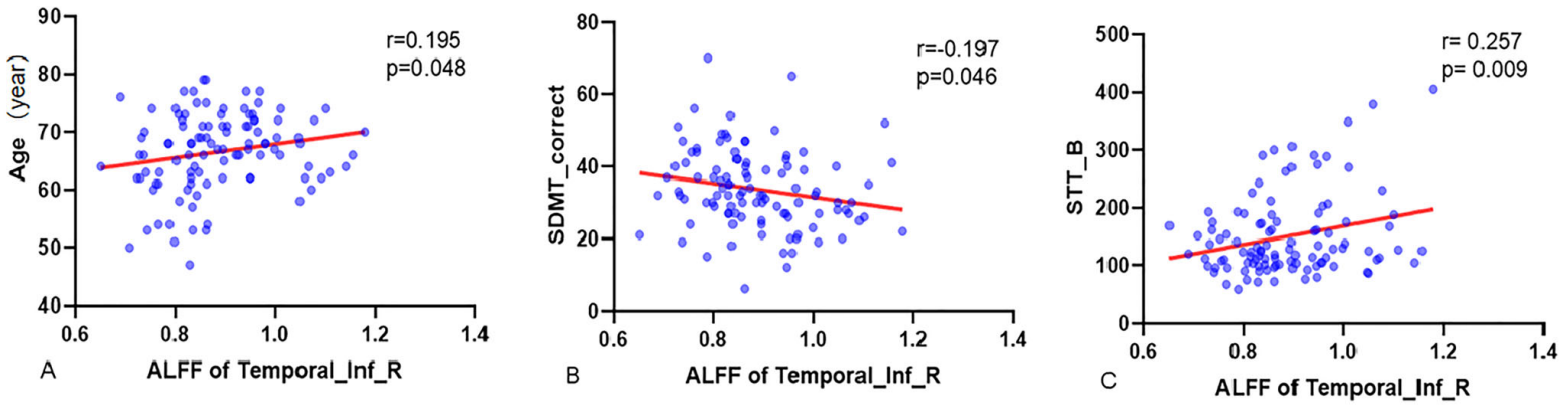
Correlation analysis between amplitude of low frequency fluctuation (ALFF) values of specific brain regions and clinical indicators in hyperlipidemia patients. (A) Positive correlation is found between age and the zALFF value of the right inferior temporal gyrus (*r* = 0.195, *p* = 0.048). (B) The relationship between SDMT and the zALFF value of the inferior temporal gyrus (*r* = −0.197, *p* = 0.046). (C) The relationship between STT‐B and the zALFF value of the right inferior temporal gyrus (*r* = 0.257, *p* = 0.009). SDMT: Symbol Digit Modalities Test; STT‐B: Shape Trail Test; Temporal_Inf_R: right inferior temporal gyrus.

## 4 Discussion

Cholesterol levels in hyperlipidemia patients are significant indicators of cognitive impairment, with a strong association between elevated cholesterol levels and accelerated cognitive decline (Goldman‐Rakic 1995). Cognitive impairment is related to aging, insomnia, and dyslipidemia, all of which affect cognitive function and may even lead to cognitive disorders (Falck et al. 2018; Okusaga et al. 2013).

### 4.1 ALFF Differences Between Groups

This study utilized rs‐fMRI to observe the correlation between functional changes in brain regions of hyperlipidemia patients and cognitive impairment. The results showed that the regions with increased zALFF values in hyperlipidemia patients were the right dorsolateral superior frontal gyrus, the right temporal pole: the middle temporal gyrus, the right inferior temporal gyrus, the right medial superior frontal gyrus, the right inferior frontal gyrus (triangular part), the right temporal pole: the superior temporal gyrus, and the right middle temporal gyrus. These regions may be related to cognitive function. These findings are consistent with earlier studies. There is substantial evidence that applying rTMS to the cognitive control network (CCN), especially the dorsolateral prefrontal cortex, can directly affect the cognitive control processes involved in emotion regulation. This indicates that applying tDCS and TMS to the dorsolateral prefrontal cortex can directly improve various mental disorders and enhance cognitive function (Amidfar et al. 2019).

The dorsolateral superior frontal gyrus, part of the frontal lobe located in the lateral prefrontal region, plays a critical role in high‐level cognitive processes and executive functions. Specifically, its primary functions include working memory, decision‐making, cognitive control, problem‐solving, reasoning, and emotional regulation (Badre and Wagner 2004; Goldman‐Rakic 1995; Miller and Cohen 2001). Cognitive impairments in hyperlipidemia patients may be related to abnormal activity in the right dorsolateral superior frontal gyrus. Studies have found that high‐fat diets leading to elevated lipid levels increase the permeability of the blood‐brain barrier, which in turn affects cognitive function (Zhang et al. 2024). Another study identified that abnormal FC in the dorsolateral inferior frontal gyrus is involved in the mechanisms of cognitive impairment, likely functioning by regulating cognitive control processes (J. Liu et al. 2023). Dyslipidemia (e.g., elevated LDL‐C) is significantly associated with global structural brain abnormalities. Although these structural changes are not strictly confined to the frontal lobe, this region—being the core hub for executive function and language processing—is highly susceptible to global structural alterations (Han et al. 2025). Dyslipidemia can lead to cognitive decline either by directly affecting large‐scale brain network reorganization or indirectly by altering cerebrospinal fluid biomarkers. Notably, 22 lipid‐related brain network connections, encompassing higher‐order cognitive networks involving the frontal lobe, have demonstrated high classification accuracy for the Alzheimer's disease (AD) spectrum (Q. Wang et al. 2022).

This study found that brain regions with increased zALFF values also included the right middle temporal gyrus, the right inferior temporal gyrus, the right temporal pole: the superior temporal gyrus, and the right temporal pole: the middle temporal gyrus, indicating enhanced spontaneous activity in these regions. The temporal lobe is a critical brain region located on both sides of the brain. It is typically divided into several important functional areas, including the superior temporal gyrus, the middle temporal gyrus, the inferior temporal gyrus, the temporal pole, the parahippocampal gyrus, the hippocampus, and the fusiform gyrus. These areas are involved in auditory processing, language processing, memory, as well as visual, and emotional processing. Previous fMRI studies have shown that the human medial temporal lobe plays a crucial role in visual object recognition, which is a key cognitive function (Y. Wang et al. 2024). The SDMT score was negatively correlated with the zALFF value of the right inferior temporal gyrus, suggesting that the right inferior temporal gyrus may be an important brain region commonly involved in the neural circuits of cognitive impairment. Furthermore, the study found a positive correlation between the STT‐B score and the zALFF value of the right inferior temporal gyrus, indicating that the overactivation of the right inferior temporal gyrus in hyperlipidemia patients might be a critical factor in the neural mechanisms underlying cognitive impairment associated with hyperlipidemia. Higher zALFF values may indicate impaired neurovascular coupling and dysregulated cerebral blood flow. As a cardiovascular risk factor, hyperlipidemia disrupts neurovascular coupling, resulting in a mismatch between cerebral blood supply and neuronal energy demands (Zimmer et al. 2025). Dyslipidemia can directly trigger cerebral network reorganization, or indirectly alter cerebrospinal fluid biomarkers to further induce cognitive decline. Accumulating studies have demonstrated that widespread brain network connectivity abnormalities driven by dyslipidemia exacerbate cognitive deterioration. Metabolic imbalance disrupts neuronal energy provision, which subsequently induces compensatory overactivation of local neural activity (Q. Wang et al. 2022). Furthermore, there is an interaction between body fat levels and cognitive status. In cognitively unimpaired (CU) individuals, higher body fat levels are significantly associated with accelerated temporal lobe atrophy, suggesting that lipid metabolism disorders may compromise the structural integrity of the temporal lobe (Fan et al. 2026).

The right medial superior frontal gyrus and the right triangular portion of the inferior frontal gyrus represent two significant anatomical areas within the frontal lobe. The right medial superior frontal gyrus: located in the central part of the frontal lobe, near the medial side of the longitudinal fissure. The right triangular part of the inferior frontal gyrus: A component of the inferior frontal gyrus, situated in the right lateral frontal lobe. Studies have shown that lesions in the frontal lobe can lead to changes in cognition and brain networks, which significantly affect cognitive functions. Research findings indicate that cognitive function can improve after surgical intervention for frontal lobe lesions, accompanied by reorganization and repair of the related brain networks (Guan et al. 2024).

### 4.2 ReHo Differences Between Groups

ReHo is a method for evaluating the consistency of neural activity within local brain regions. It measures the characteristics of brain activity by comparing the similarity of neural responses among adjacent voxels. The cerebellum has garnered significant attention due to its involvement in regulating the activity of extensive brain regions and its critical roles in motor function, cognition, and emotional behavior. The cerebellum plays an essential role in cognitive processes. In this study, we observed that the excitability (ReHo) of affected cerebellar regions was significantly reduced in the hyperlipidemia group, indicating that hyperlipidemia contributes to abnormalities in brain function. Based on this, we conducted a correlation analysis between brain activation and neuropsychological clinical scales. Our findings revealed no significant correlation between changes in cerebellar activation levels (ReHo) and clinical scales in the hyperlipidemia group. Previous studies have shown that cerebellar infarction may result in cognitive impairment. However, compared to patients with frontal lobe infarction, those with cerebellar infarction experience relatively milder cognitive impairment, suggesting that the cerebellum influences cognitive function and can contribute to cognitive dysfunction (Liu and Zhang 2024). Research has confirmed, through physiological and anatomical studies, the organizational structure of the cerebellum, including separate motor areas and distinct cognitive brain regions involved (Takla et al. 2025). This underscores the cerebellum's close relationship with our cognitive processes. Regarding the cerebellum, distinct lobules exhibit variations in lipid and protein distribution. The close relationship between its structural development and functional characteristics during childhood suggests that local cerebellar lipid metabolism plays a fundamental role in maintaining normal neurological function (X. Liu et al. 2022). Beyond motor coordination, the cerebellum is also involved in non‐motor functions such as cognition and emotional processing. Hyperlipidemia‐induced neuroinflammation and oxidative stress may disrupt cerebellar‐cortical circuits, thereby impairing its compensatory role in various neurological disorders, including AD and Parkinson's disease (Sivalingam 2024).

### 4.3 FC Differences Between Groups

Seed‐based FC is a commonly used method for analyzing functional connections, allowing us to investigate the correlation between the FC of a region of interest and other brain regions (Damoiseaux et al. 2006). Our results indicate that, compared to the healthy control group, the hyperlipidemia group showed significantly enhanced FC between the dorsolateral superior frontal gyrus and the fusiform gyrus, the superior cerebellum, and the middle temporal gyrus. Both the cerebellum and the temporal lobe are associated with cognition, while the fusiform gyrus, located on the inferior side of the temporal and occipital lobes, plays a critical role in various advanced visual and cognitive functions. Notably, the fusiform gyrus is prominently involved in reading and word recognition (McCandliss et al. 2003). The abnormally increased FC in hyperlipidemia patients suggests an over‐coupling of the network between the dorsolateral superior frontal gyrus and the fusiform gyrus, the superior cerebellum, and the middle temporal gyrus, which may result in reduced information processing efficiency. This indicates that in hyperlipidemia patients, the dorsolateral superior frontal gyrus is more tightly connected to the fusiform gyrus, the superior cerebellum, and the middle temporal gyrus compared to healthy individuals. Abnormal lipid profiles (elevated LDL‐C, reduced HDL) are associated with whole‐brain network remodeling, which may degrade interregional FC spanning the frontal–temporal–cerebellar circuit axis (Q. Wang et al. 2022).

Prior observational studies link elevated cardiovascular risk burden to attenuated FC within discrete cognitive networks, supporting cerebrovascular damage as a key intermediate pathway for connectivity dysfunction (Chauveau et al. 2025). In certain pathological conditions, decreased cerebrocerebellar FC is accompanied by enhanced cortico‐cortical network connectivity. This reflects a compensatory redistribution at the network level, suggesting that hyperlipidemia‐related brain damage may trigger similar adaptive reorganization (Park et al. 2022).

## 5 Limitations

This research has multiple limitations. First, the sample size was comparatively small, affecting the generalizability of findings, which may introduce some bias in the results. In addition, no subgroup analysis was conducted to compare different types of hypercholesterolemia. Future research could expand the sample size and further investigate brain activity differences between patients with familial hypercholesterolemia and those with non‐familial hypercholesterolemia.

The lower SDMT score observed in the hyperlipidemia group should be interpreted cautiously. Although the SDMT is sensitive to information processing speed, performance on this test also depends on attention, visual scanning, and working memory. Because the present study included only one measure primarily targeting processing speed and did not prespecify processing speed as the primary cognitive endpoint, the findings cannot establish a selective impairment in this domain. Moreover, the association between SDMT performance and the right inferior temporal gyrus zALFF was modest and uncorrected for multiple comparisons. It should therefore be regarded as exploratory and requires confirmation using a comprehensive processing‐speed battery and an independent sample.

Following correlation analyses and multiple comparison correction, correlations between imaging metrics and clinical scale scores did not survive. This may be explained by two factors: the relatively small sample size and inherent variability within imaging measurements. Therefore, correlational trends derived from uncorrected analyses should not be overinterpreted, as multiple testing is prone to generating spurious statistical associations. Future studies should mitigate statistical bias from pairwise comparisons, enroll larger samples, and construct multivariate analytical models.

## 6 Conclusion

This study identified lower SDMT performance and altered spontaneous brain activity and functional connectivity in frontal, temporal, and cerebellar regions in patients with hyperlipidemia. Exploratory analyses suggested that activity in the right inferior temporal gyrus may be associated with SDMT and STT‐B performance. These findings provide preliminary evidence of a relationship between hyperlipidemia‐related functional brain alterations and cognitive performance, but they do not establish a specific neural biomarker of information processing speed.

## Author Contributions

**Jian‐Hui He**: conceptualization, writing – original draft. **Jia‐Jia Wu**: conceptualization. **Fan‐Long Meng**: investigation. **Yun‐Ting Xiang**: formal analysis. **Rong‐Rong Huang**: validation. **Mou‐Xiong Zheng**: writing – review and editing, supervision. **Jie Ma**: visualization, data curation, formal analysis. **Jian‐Guang Xu**: writing – review and editing, supervision. All authors have read and approved the final manuscript.

## Funding

This work was supported by the National Natural Science Foundation of China (grant nos.: 82272583, 82172554, 82272589, 82302870, 82472613, 82472589, 82402991), the Shanghai Municipal Health Commission Collaborative Innovation Group (grant no.: 2024CXJQ03), the Shanghai Health Care Commission (grant no.: 2022JC026), the Shanghai Science and Technology Committee (grant no.: 22010504200), the Shanghai Municipal Special Fund for Promoting High‐Quality Industrial Development (grant no.: 240404), the Shanghai Innovative Medical Device Application Demonstration Project (grant no.: 23SHS05600); the Shanghai Rising‐Star Program (grant nos.: 23QA1409200, 24QA2709300), the Shanghai Oriental Talents Program (grant nos.: QNWS2024084, QNJY2024077), the Shanghai University of Traditional Chinese Medicine Science and Technology Development Fund (grant no.: 23KFL112), the Shanghai Talent Development Fund (grant no.: 2021074), the High‐level Chinese Medicine Key Discipline Construction Project (Integrative Chinese and Western Medicine Clinic) of the National Administration of TCM (grant no.: zyyzdxk‐2023065), and the Shanghai Hospital Development Center Foundation‐Shanghai Municipal Hospital Rehabilitation Medicine Specialty Alliance (grant no.: SHDC22023304).

## Ethics Statement

The research, which involves human participants, has received formal approval from the Institutional Review Board (IRB) at Yueyang Hospital of Integrated Traditional Chinese and Western Medicine, affiliated with the Shanghai University of Traditional Chinese Medicine, ensuring that all ethical standards are met (No. 2019‐124). The consent that was obtained from all of the participants was informed.

## Conflicts of Interest

The authors declare no conflicts of interest.

## Data Availability

The raw data supporting the conclusions of this article will be accessible by the authors upon request, ensuring transparency and availability for further review and verification.

## References

[brb371890-bib-0001] Amidfar, M. , Y. H. Ko , and Y. K. Kim . 2019. “Neuromodulation and Cognitive Control of Emotion.” Advances in Experimental Medicine and Biology 1192: 545–564. 10.1007/978-981-32-9721-0_27. 31705513

[brb371890-bib-0002] Asgary, S. , L. Salehizadeh , M. Keshvari , et al. 2018. “Potential Cardioprotective Effects of Sumac Capsule in Patients With Hyperlipidemia: A Triple‐Blind Randomized, Placebo‐Controlled Crossover Trial.” Journal of the American College of Nutrition 37, no. 4: 286–292. 10.1080/07315724.2017.1394237. 29425477

[brb371890-bib-0003] Badre, D. , and A. D. Wagner . 2004. “Selection, Integration, and Conflict Monitoring; Assessing the Nature and Generality of Prefrontal Cognitive Control Mechanisms.” Neuron 41, no. 3: 473–487. 10.1016/s0896-6273(03)00851-1. 14766185

[brb371890-bib-0004] Can, H. , E. Doğutepe , N. Torun‐Yazıhan , H. Korkman , and E. Erdoğan Bakar . 2016. “Construct Validity of Auditory Verbal Learning Test.” Turk Psikiyatri Dergisi 27, no. 3: 195–203. 27711940

[brb371890-bib-0005] Chauveau, L. , J. Gonneaud , G. Poisnel , et al. 2025. “Cardiovascular Risk Factors Are Associated With Lower Posterior‐Medial Network Functional Connectivity in Older Adults.” Alzheimer's Research & Therapy 17, no. 1: 159. 10.1186/s13195-025-01808-5. PMC12261594 40665410

[brb371890-bib-0006] Chen, H. , K. Jann , Y. Li , et al. 2023. “A True Response of the Brain Network During Electroacupuncture Stimulation at Scalp Acupoints: An fMRI With Simultaneous EAS Study.” Brain and Behavior 13, no. 1: e2829. 10.1002/brb3.2829. PMC9847615 36427258

[brb371890-bib-0007] Chou, M. C. , H. C. Lee , Y. C. Liu , et al. 2022. “Long‐Term High‐Fat Diet Consumption Depletes Glial Cells and Tyrosine Hydroxylase–Containing Neurons in the Brain of Middle‐Aged Rats.” Cells 11, no. 2: 295. 10.3390/cells11020295. PMC8773849 35053411

[brb371890-bib-0008] Damoiseaux, J. S. , S. A. Rombouts , F. Barkhof , et al. 2006. “Consistent Resting‐State Networks Across Healthy Subjects.” Proceedings of the National Academy of Sciences 103, no. 37: 13848–13853. 10.1073/pnas.0601417103. PMC1564249 16945915

[brb371890-bib-0009] Deng, Y. Y. , X. Zhou , B. Y. Yin , et al. 2026. “Neuroimaging Biomarkers in School‐Aged Children With Autism: MRI‐Measured Lipid Content in the Limbic System.” Journal of Child Psychology and Psychiatry 67, no. 5: 631–640. 10.1111/jcpp.70033. 40791070

[brb371890-bib-0010] Falck, R. S. , J. R. Best , L. C. Li , P. C. Y. Chan , L. M. Feehan , and T. Liu‐Ambrose . 2018. “Can We Improve Cognitive Function Among Adults With Osteoarthritis by Increasing Moderate‐to‐Vigorous Physical Activity and Reducing Sedentary Behaviour? Secondary Analysis of the MONITOR‐OA Study.” BMC Musculoskeletal Disorders 19, no. 1: 447. 10.1186/s12891-018-2369-z. PMC6303889 30577819

[brb371890-bib-0011] Fan, L. , Y. Sun , D. Liu , et al. 2026. “High‐Fat Diet Is Associated With Accelerated Gray Matter Atrophy in Cognitively Unimpaired Older Adults but Slower Atrophy in Individuals With Existing Mild Cognitive Impairment.” Alzheimer's & Dementia 22, no. 6: e71548. 10.1002/alz.71548. PMC13253361 42271188

[brb371890-bib-0012] Fellows, R. P. , and M. Schmitter‐Edgecombe . 2019. “Symbol Digit Modalities Test: Regression‐Based Normative Data and Clinical Utility.” Archives of Clinical Neuropsychology 35, no. 1: 105–115. 10.1093/arclin/acz020. 31329813

[brb371890-bib-0013] Goldman‐Rakic, P. S. 1995. “Cellular Basis of Working Memory.” Neuron 14, no. 3: 477–485. 10.1016/0896-6273(95)90304-6. 7695894

[brb371890-bib-0014] Guan, X. , B. Hu , W. Zheng , et al. 2024. “Short‐Term Changes in Brain Networks and Cognition in Children With Frontal Lobe Lesions Treated Solely With Neurosurgical Procedures.” Scientific Reports 14, no. 1: 29930. 10.1038/s41598-024-73088-2. PMC11611908 39622933

[brb371890-bib-0015] Han, L. , Q. Li , L. Zhang , et al. 2025. “The Necessity of Strengthening Glycemic and Lipid Metabolism Management for Improving Brain Structure and Cognitive Function in People With Diabetes: A Retrospective Study Based on UK Biobank.” Diabetes Research and Clinical Practice 226: 112366. 10.1016/j.diabres.2025.112366. 40659220

[brb371890-bib-0016] Li, X. , W. Zhang , Y. Bi , et al. 2024. “Non‐Alcoholic Fatty Liver Disease Is Associated With Brain Function Disruption in Type 2 Diabetes Patients Without Cognitive Impairment.” Diabetes, Obesity and Metabolism 26, no. 2: 650–662. 10.1111/dom.15354. 37961040

[brb371890-bib-0017] Li, Y. , X. Tian , Z. Y. Xiong , et al. 2016. “Performance of the Modified Mini‐Mental State Examination (3MS) in Assessing Specific Cognitive Function in Patients Undergoing Peritoneal Dialysis.” PLoS ONE 11, no. 12: e0166470. 10.1371/journal.pone.0166470. PMC5135044 27911914

[brb371890-bib-0018] Liu, J. , X. Zou , J. Gu , et al. 2023. “Altered Connectivity in the Cognitive Control‐Related Prefrontal Cortex in Parkinson's Disease With Rapid Eye Movement Sleep Behavior Disorder.” Brain Imaging and Behavior 17, no. 6: 702–714. 10.1007/s11682-023-00796-0. 37721659

[brb371890-bib-0019] Liu, Q. , and Y. Zhang . 2024. “A Comparative Study on Cognitive Assessment in Cerebellar and Supratentorial Stroke.” Brain Sciences 14, no. 7: 676. 10.3390/brainsci14070676. PMC11274804 39061417

[brb371890-bib-0020] Liu, X. , F. d'Oleire Uquillas , A. N. Viaene , Z. Zhen , and J. Gomez . 2022. “A Multifaceted Gradient in Human Cerebellum of Structural and Functional Development.” Nature Neuroscience 25, no. 9: 1129–1133. 10.1038/s41593-022-01136-z. 35982153

[brb371890-bib-0021] McCandliss, B. D. , L. Cohen , and S. Dehaene . 2003. “The Visual Word Form Area: Expertise for Reading in the Fusiform Gyrus.” Trends in Cognitive Sciences 7, no. 7: 293–299. 10.1016/s1364-6613(03)00134-7. 12860187

[brb371890-bib-0022] Miller, E. K. , and J. D. Cohen . 2001. “An Integrative Theory of Prefrontal Cortex Function.” Annual Review of Neuroscience 24: 167–202. 10.1146/annurev.neuro.24.1.167. 11283309

[brb371890-bib-0023] Okusaga, O. , M. C. Stewart , I. Butcher , I. Deary , F. G. Fowkes , and J. F. Price . 2013. “Smoking, Hypercholesterolaemia and Hypertension as Risk Factors for Cognitive Impairment in Older Adults.” Age and Ageing 42, no. 3: 306–311. 10.1093/ageing/afs193. 23302603

[brb371890-bib-0024] Park, H. R. , J. Cha , E. Y. Joo , and H. Kim . 2022. “Altered Cerebrocerebellar Functional Connectivity in Patients With Obstructive Sleep Apnea and Its Association With Cognitive Function.” Sleep 45, no. 1: zsab209. 10.1093/sleep/zsab209. PMC8754484 34432059

[brb371890-bib-0025] Rodriguez‐Seijas, C. , J. S. Thompson , J. M. Diehl , and M. Zimmerman . 2020. “A Comparison of the Dimensionality of the Hamilton Rating Scale for Anxiety and the DSM‐5 Anxious‐Distress Specifier Interview.” Psychiatry Research 284: 112788. 10.1016/j.psychres.2020.112788. 31978629

[brb371890-bib-0026] Sivalingam, A. M. 2024. “Advances in Understanding Biomarkers and Treating Neurological Diseases—Role of the Cerebellar Dysfunction and Emerging Therapies.” Ageing Research Reviews 101: 102519. 10.1016/j.arr.2024.102519. 39341507

[brb371890-bib-0027] Takla, T. N. , J. Feldpausch , E. M. Edwards , et al. 2025. “Cerebellar Volume Measures Differentiate Multiple Sclerosis Fallers from Non‐Fallers.” Brain Sciences 15: 1. 10.3390/brainsci15010077. PMC11764211 39851444

[brb371890-bib-0028] Thorvaldsson, V. , I. Skoog , and B. Johansson . 2020. “Cholesterol and Cognitive Aging: Between‐Person and Within‐Person Associations in a Population‐Based Representative Sample Not on Lipid‐Lowering Medication.” Psychology and Aging 35, no. 4: 508–516. 10.1037/pag0000449. 32105111

[brb371890-bib-0029] Tombaugh, T. N. , and A. M. Hubiey . 1997. “The 60‐Item Boston Naming Test: Norms for Cognitively Intact Adults Aged 25 to 88 Years.” Journal of Clinical and Experimental Neuropsychology 19, no. 6: 922–932. 10.1080/01688639708403773. 9524887

[brb371890-bib-0030] Tombaugh, T. N. , and N. J. McIntyre . 1992. “The Mini‐Mental State Examination: A Comprehensive Review.” Journal of the American Geriatrics Society 40, no. 9: 922–935. 10.1111/j.1532-5415.1992.tb01992.x. 1512391

[brb371890-bib-0031] Wang, Q. , F. Zang , C. He , Z. Zhang , and C. Xie . 2022. “Dyslipidemia Induced Large‐Scale Network Connectivity Abnormality Facilitates Cognitive Decline in the Alzheimer's Disease.” Journal of Translational Medicine 20, no. 1: 567. 10.1186/s12967-022-03786-w. PMC9724298 36474263

[brb371890-bib-0032] Wang, T. J. , A. S. Lien , J. L. Chen , C. H. Lin , Y. S. Yang , and S. H. Yang . 2019. “A Randomized Clinical Efficacy Trial of Red Yeast Rice (*Monascus pilosus*) Against Hyperlipidemia.” American Journal of Chinese Medicine 47, no. 2: 323–335. 10.1142/s0192415x19500150. 30871361

[brb371890-bib-0033] Wang, Y. , R. Cao , and S. Wang . 2024. “Encoding of Visual Objects in the Human Medial Temporal Lobe.” Journal of Neuroscience 44, no. 16: e2135232024. 10.1523/jneurosci.2135-23.2024. PMC11026346 38429107

[brb371890-bib-0034] Yoon, H. , L. F. Flores , and J. Kim . 2016. “MicroRNAs in Brain Cholesterol Metabolism and Their Implications for Alzheimer's Disease.” Biochimica et Biophysica Acta (BBA)—Molecular and Cell Biology of Lipids 1861, no. 12 Pt B: 2139–2147. 10.1016/j.bbalip.2016.04.020. PMC5097035 27155217

[brb371890-bib-0035] Zang, Y. , T. Jiang , Y. Lu , Y. He , and L. Tian . 2004. “Regional Homogeneity Approach to fMRI Data Analysis.” NeuroImage 22, no. 1: 394–400. 10.1016/j.neuroimage.2003.12.030. 15110032

[brb371890-bib-0036] Zhang, M. , Y.‐H. Zhu , and Z.‐Q. Zhu . 2024. “Research Advances in the Influence of Lipid Metabolism on Cognitive Impairment.” Ibrain 10, no. 1: 83–92. 10.1002/ibra.12018. PMC11045198 38682015

[brb371890-bib-0037] Zhao, Q. , Q. Guo , F. Li , Y. Zhou , B. Wang , and Z. Hong . 2013. “The Shape Trail Test: Application of a New Variant of the Trail Making Test.” PLoS ONE 8, no. 2: e57333. 10.1371/journal.pone.0057333. PMC3577727 23437370

[brb371890-bib-0038] Zhao, Y. , H. Zhang , J. Cheng , Y. Zou , D. Zhang , and X. Duan . 2024. “Association Between Dyslipidaemia and Cognitive Impairment: A Meta‐Analysis of Cohort and Case‐Control Studies.” Journal of Integrative Neuroscience 23, no. 2: 40. 10.31083/j.jin2302040. 38419448

[brb371890-bib-0039] Zimmer, A. R. , M. Bourourou , F. Lesage , and E. Hamel . 2025. “Neurovascular Coupling and Functional Connectivity Changes Through the Alzheimer's Disease Spectrum: Effects of Simvastatin Treatment.” Alzheimer's & Dementia 21, no. 8: e70402. 10.1002/alz.70402. PMC12314550 40747637

[brb371890-bib-0040] Zimmerman, M. , J. H. Martinez , D. Young , I. Chelminski , and K. Dalrymple . 2013. “Severity Classification on the Hamilton Depression Rating Scale.” Journal of Affective Disorders 150, no. 2: 384–388. 10.1016/j.jad.2013.04.028. 23759278

